# Tact

**Published:** 1884-04

**Authors:** 


### TACT

PEOPLE cannot help having been born without tact, any more than they can help having no ear for music; but there are occasions when it is almost impossible to be quite charitable to a tactless person. Yet people who have no tact deserve pity. They are almost always doing or saying something to get themselves into disgrace. or which does them an injury. They make enemies when they desire friends, and get a reputation for ill-nature which they do not deserve. They are also continually doing other people harm, treading on metaphorical corns, opening the cupboards where family skeletons are kept, angering people, shaming people, saying and doing the most awkward things, and apologizing for them with a still more terrible bluntness. If there is one social boon more to be desired than another. it is tact; for, without tact, the career of the richest and most beautiful is often utterly ruined.

